# Burning Mouth Syndrome Induced by Angiotensin-Converting Enzyme Inhibitors

**DOI:** 10.7759/cureus.11376

**Published:** 2020-11-08

**Authors:** Takafumi Obara, Hiromichi Naito, Tsuyoshi Nojima, Hitoshi Koga, Atsunori Nakao

**Affiliations:** 1 Emergency, Critical Care, and Disaster Medicine, Okayama University Graduate School of Medicine, Dentistry and Pharmaceutical Sciences, Okayama, JPN; 2 Emergency Medicine, St. Mary Hospital, Kurume, Fukuoka, JPN

**Keywords:** burning mouth syndrome, oral mucosa, ace inhibitor, pain management

## Abstract

Burning mouth syndrome (BMS) is characterized as the sensation of burning in the tongue or any other area of the oral mucosa without a medical or dental cause. BMS's pathophysiology is poorly understood and may be caused by its association with various factors, particularly with antihypertensive or psychotropic medicines. Although BMS is a relatively common intraoral disorder in the dental field, emergency physicians rarely recognize it. We report a 53-year-old Japanese woman who presented to our ED with a three-week history of a strange taste and burning tongue. The patient had poor control of hypertension, captopril, an angiotensin-converting enzyme (ACE) inhibitor, was added three weeks before presentation. We discontinued her ACE inhibitor and changed her medication to a beta-blocker. After two weeks, her symptoms improved. Emergency physicians must be aware that ACE inhibitors play some roles in the pathogenesis of BMS. The correct diagnosis of the condition depends on recognizing the disease and improving the quality of life.

## Introduction

Burning mouth syndrome (BMS) is characterized as the sensation of burning in the tongue or any other area of the oral mu­cosa without a medical or dental cause [1]. The pathogenesis of BMS has not been fully elucidated; however, associations with several factors of varying origins, including psychological and biological, have been implicated.

Here, we describe a patient who developed BMS after initiation of angiotensin-converting enzyme (ACE) inhibitors for hypertension whose oral symptoms were eliminated by discontinuing ACE inhibitors and changing to a beta-blocker medication. An association between the onset of a burning sensation in the mouth and the administration of drugs has been known, particularly with antihypertensive or psychotropic medicines [2-5]. Although BMS is a relatively common intraoral disorder in the dental field, it is rarely recognized by emergency physicians. However, the condition has a significant impact on the quality of life. This article’s purpose is to raise awareness as well as review the literature of this rare occurrence.

## Case presentation

A 53-year-old Japanese woman presented to our ED with a three-week history of a strange taste and burning tongue. These symptoms resulted in loss of appetite, sleep disturbance, and general fatigue. She had no history of drug or alcohol abuse. Her past medical history was positive for hypertension, which was maintained on amlodipine besylate 5 mg/day. Captopril, an ACE inhibitor, was added three weeks before presentation due to poor control of hypertension. 

When examined, the patient seemed healthy, cooperative, conscious, well-oriented, and well-nourished. Her vital signs were normal, with a blood pressure of 134/88 mmHg and a heart rate of 74 beats/minute. Intraoral examination revealed normal and healthy oral mucosa without mucosal lesions such as aphthae or tumors to explain the pain. Her salivary secretion seemed adequate. The patient did not report symptoms associated with depression syndrome. A review of her laboratory test results revealed normal serum chemistry profile and blood counts. Additionally, her serum iron, zinc, and vitamin B12 levels were normal.

The patient was referred to a local dental clinic and examined by a dentist, and she was diagnosed with BMS. Subsequently, the patient was referred to a psychiatric clinic and alprazolam treatment was initiated. However, her oral symptoms did not lessen but rather gradually increased over the following weeks despite anti-anxiety medications. Since her symptoms could be a side effect of ACE inhibitors, we discontinued her ACE inhibitor and changed her medication to a beta-blocker. After two weeks, her symptoms improved. Her taste perception returned to normal, the burning sensation stopped, her appetite increased, and her mood was stable. Currently, her oral mucosa, conjunctiva, and tongue appear normal and she is not experiencing any oral symptoms. Follow-up has not revealed any evidence of underlying medical or neurological disorders.

## Discussion

The manifestations of BMS are described as a burning feeling; pain, discomfort, and rawness or irritation of the lips, tongue, or oral cavity; as well as other associated symptoms such as unusual sensations, taste alteration, appetite loss, and general fatigue. According to the third edition of the International Classification of Headache Disorders (ICHD-3), BMC is generally defined as “An intraoral burning or dysaesthetic sensation, recurring daily for more than two hours/day over more than three months, without clinically evident causative lesions” [6]. It typically affects elderly postmenopausal women with a prevalence of up to 12%-33% [7-8]. Despite its significance, BMS tends to receive relatively little attention from medical professionals and is underreported by patients, who may not link these effects with drug therapy [9]. The complaints of patients with BMS are sometimes considered nonspecific and subsequently, these patients are undertreated.

Burning mouth syndrome can be potentially primary or secondary to other conditions including local, systemic, and psychological diseases. Local BMS causes include food allergens, candidiasis, and potential friction on the oral mucosa from dentures. Systemic factors include iron-deficiency anemia, smoking, menopause, diabetes mellitus, Sjogren’s syndrome, postzoster neuropathy, allergy, nutrient deficiency, and medications like antidepressants and diuretics. Psychogenic factors involved in the development of BMS are anxiety, depression, or personality disorders, and could be related to dopaminergic hypofunction.

In our patient, the onset of BMS symptoms was closely correlated to initiation of administration of an ACE inhibitor for hypertension. Also, stopping the ACE inhibitor resulted in alleviation of the symptoms within two weeks. These associations of BMS with ACE inhibitors suggested that BMS may be an adverse effect of ACE inhibitors. BMS is an infrequently reported adverse effect of captopril treatment and is even less frequently reported with other ACE inhibitors that act upon the angiotensin-renin system [2, 9-10]. These adverse effects have been reported to be attributed to the chemical structure of the drugs, although this relationship is the matter of some debate [9]. A conflict exists between BMS’s association with a wide variety of drugs and the observation of large differences among medications of the same class, indicating that BMS is not likely to be related to a pharmacological effect [5, 11]. Thus, evidence for this relationship is still equivocal. The onset of BMS after use of ACE inhibitor is not currently fully understood, and the reason for the occurrence of this side effect after long-term problem-free use remains unexplained. In the previous reports, the interval between an initiation of ACE inhibitor and onset of BMS varied ranging from 6 days to 7 years [5]. BMS might be related to dose, and pharmacodynamic and physiological changes are expected in many aging patients. However, the pathogenesis of BMS has not been fully elucidated.

Recent neuropathological, neurophysiological, psychophysical, and functional imaging studies have revealed that various neuropathic mechanisms, mostly subclinical, act at different neuraxial levels and contribute to the pathophysiology of BMS. Studies of superficial tongue biopsy demonstrated that BMS is caused by a trigeminal small-fiber sensory neuropathy [12]. Presynaptic nigrostriatal dopaminergic pathway dysfunction may contribute to chronic pain in BMS [13]. Immunohistochemical studies of biopsied specimens of tongues of BMS patients revealed a significant correlation between pain score and the heat and capsaicin receptor transient receptor potential vanilloid 1 (TRPV1), as well as its regulator nerve growth factor [14].

Identification of the underlying etiology is the goal for and treatment of BMS. The determination of the exact etiology can be achieved after other potential causes are ruled out. Thus, basically, BMS is a diagnosis of exclusion. If the patient takes an ACE inhibitor, drug cessation is the best way to treat symptoms after consulting a physician for hypertension management. Various vitamins and minerals, saliva substitutes, and hormone replacement therapy have been attempted with little success. As BMS was highly associated with anxiety disorders, the mainstay in the BMS treatment includes benzodiazepines, antidepressants, and anticonvulsants [1]. However, we must be aware that anxiolytic drugs play some roles in the pathogenesis of BMS, which may cause a dilemma in management [4].

## Conclusions

In conclusion, our patient and previous literature reviews may suggest a high probability of an association between ACE inhibitors and BMS onset.
